# Pharmacogenetic predictors of nausea and vomiting of pregnancy severity and response to antiemetic therapy: a pilot study

**DOI:** 10.1186/1471-2393-13-132

**Published:** 2013-06-20

**Authors:** Amalia S Lehmann, Jamie L Renbarger, Catherine L McCormick, Ariel R Topletz, Carrie Rouse, David M Haas

**Affiliations:** 1 Division of Clinical Pharmacology, Indiana University School of Medicine, Indianapolis, IN, USA; 2 Department of Pediatrics, Indiana University School of Medicine, Indianapolis, IN, USA; 3 Department of Obstetrics and Gynecology, Indiana University School of Medicine, Indianapolis, IN, USA

**Keywords:** Pregnancy, Nausea and vomiting of pregnancy, Antiemetics, Pharmacogenomics

## Abstract

**Background:**

Nausea and vomiting of pregnancy (NVP) is a common condition. The objective of this study was to evaluate the association between response to antiemetics in the treatment of NVP and genetic polymorphisms in the serotonin receptor subunit genes *HTR3A* and *HTR3B*.

**Methods:**

Pregnant women ≥18 years of age with NVP starting antiemetic therapy with promethazine, prochlorperazine, metoclopramide, or ondansetron at ≤ 16 weeks gestational age were eligible. The study recruited 29 women with complete data and sampling who returned for their one week follow-up and were genotyped for *HTR3A* and *HTR3B* polymorphisms. Severity of NVP was captured (using Pregnancy Unique Quantification of Emesis (PUQE) and Quality of Life (QOL) tools) upon enrollment and after one week of antiemetic therapy. These measures were correlated with pharmacogenetic variability.

**Results:**

Subjects with genotype associated with high serotonin affinity of the 5-HT_3B_ receptor (rs1176744, CC) required more antiemetic medications (p < 0.001) than other subjects. Those with genotypes associated with increased expression of the 5-HT_3A_ receptor subunit (rs1062613, CT or TT) had worse final PUQE scores (p = 0.01) than other subjects while rs3782025 variants carriers had significantly better initial (p = 0.02) and final (p = 0.02) PUQE scores than other subjects.

**Conclusions:**

*HTR3B* and *HTR3A* gene variants may contribute to variability in response to antiemetic therapy for NVP.

## Background

Nausea and vomiting of pregnancy (NVP) affects up to 80% of all pregnant women [1-3]. Although NVP is most common during the first trimester of pregnancy, as many as 20% of pregnant women are affected beyond 12 weeks of gestation and throughout the day (vs. isolated to the morning hours). Of the women affected, 1 to 3% experience a severe form of NVP called hyperemesis gravidarum (HG) which includes weight loss, dehydration, and electrolyte imbalances. In mild cases, NVP causes discomfort and inconvenience; however, HG can pose significant risks to the health of the pregnant woman and the fetus—often necessitating hospitalization [4]. Even the milder cases of NVP can have a significant impact on the quality of a woman’s life and contribute significantly to health care costs and time lost from work [2,3,5]. Termination of otherwise wanted pregnancies has been reported in women suffering from severe, prolonged NVP [6]. However, because NVP is rarely life-threatening, the goal of pharmacologic treatment is typically improvement in the quality of life for affected pregnant women [7].

Several dopamine antagonist antiemetics commonly used in pregnancy also act as weak serotonin receptor antagonists. These include promethazine, prochlorperazine, and metoclopramide. Additionally, the 5-HT_3_ receptor antagonist ondansetron is an often used antiemetic in pregnancy. These antiemetics have been studied extensively and all seem to be safe for use in pregnancy [7-10]. However, response to these medications in the treatment of NVP is highly variable [7,11-13]. While pharmacologic intervention may ameliorate symptoms in many women, some women improve dramatically while others continue to experience severe NVP despite treatment. As such, pharmacogenetic polymorphisms become an important consideration in evaluating variability in responsiveness to pharmacologic intervention. Many antiemetics are substrates for serotonin receptors and transporters—which are known to have clinically relevant genetic polymorphisms. Previous studies have shown that variations in the *HTR3B* gene predict the efficacy of antiemetics in cancer patients [14] and the occurrence of selective serotonin reuptake inhibitor (SSRI)-induced nausea [15]. However, there have been limited studies which have investigated the effects of the serotonin receptor gene polymorphisms on the efficacy of antiemetics used in the treatment of NVP [16].

The objective of this study was to evaluate the role of serotonin receptor subunit *HTR3A* and *HTR3B* genotype with NVP severity and antiemetic efficacy.

## Methods

### Study population

Pregnant women ≥18 years of age diagnosed with NVP during the first trimester of pregnancy were recruited from obstetric clinics associated with Indiana University School of Medicine. Subjects had to be experiencing NVP for at least one week. Subjects were recruited when presenting for therapy for NVP to either an acute care area or prenatal clinic. Informed consent was obtained from all subjects. Women with multiple gestations or beyond 16 weeks gestation were excluded. Women with a history of hypertension, epilepsy, or diabetes; history of hepatitis or gastrointestinal disorders that might manifest as nausea or vomiting; history of hyperthyroidism or current hyperthyroidism; or who were unwilling to complete a follow-up visit within one week or to complete NVP severity surveys over the telephone were excluded from the study.

Subject demographics, gestational age at presentation, and the medications prescribed by the provider were all recorded. As this was not a treatment trial, the drug therapy choice was left to the treating provider. The study was approved by the Indiana University Purdue University-Indianapolis Institutional Review Board.

### Sample collection and processing

A collection of up to 10 mL of whole blood in a K_2_EDTA vacutainer was obtained for DNA sampling and pharmacogenetic testing upon enrollment in the study. Samples were stored at −80°C until DNA isolation was completed. If a whole blood sample could not be obtained from the enrolled patient for any reason a saliva sample was taken for DNA sampling and pharmacogenetic testing using an Oragene® saliva kit collection.

DNA was extracted from whole blood samples using the QIAamp® DNA mini kits (Qiagen Inc., Valencia, CA). Manufacturer spin protocol instructions were followed. Isolation of DNA from saliva samples was done following the manufacturer specified protocol “Laboratory Protocol for Manual Purification of DNA from Oragene® DNA/Saliva.” All DNA samples were transferred into cryovials and stored at −80°C until quantification. Concentration of double-stranded DNA in our samples was determined using a Quant-iT dsDNA Broad Range or High Sensitivity Assay Kit and Qubit Flourometer (Invitrogen, Carlsbad, California.)

### Pharmacogenetics

Subject samples were genotyped for the following serotonin receptor gene SNPs: *HTR3B* nonsynonymous SNP Tyr129Ser (rs1176744); *HTR3B* intronic SNP (rs3782025); *HTR3B* deletion −104_-102delAGA (also known by the alias −102_-100delAAG) (rs3831455); and *HTR3A* 5’-UTR SNP Pro16Ser (rs1062613). Genotyping was done using predesigned or custom commercially available Taqman Real-Time polymerase chain reaction (PCR) assays following manufacturer published methods (Applied Biosystems, Darmstadt, Germany). Although our genes of interest encode hundreds of amino acids, we thought it most appropriate in this pilot study to select several SNPs that had previously been cited as having functional and/or clinical significance in afflictions largely characterized by nausea and vomiting. Additionally, these SNPs were chosen based on commercial availability and population prevalence. Based on the available population prevalence data, all chosen SNPs showed a minor allele frequency of at least 5% in all ethnic groups that participated in this study. For instance the minor allele frequency in European and African populations for rs1176744 are 0.29 and 0.43, respectively. Rs 3782025 has minor allele frequencies of 0.46 and 0.40 in the same populations respectively and rs 1062613 has minor allele frequencies of 0.21 and 0.49 in the same populations, respectively (http://www.ncbi.nlm.nih.gov accessed 8/8/2012).

### NVP severity tools

The Motherisk Pregnancy Unique Quantification of Emesis (PUQE) score, a validated scoring tool (Table 1) to quantify the severity of NVP [17,18], was used to assess self-perceived symptom severity and well-being. Based on quantification of the three physical symptoms of NVP, PUQE closely correlates with the Rhodes’ Index score, another validated but much more complex tool. To further assess specific ways in which NVP impacts women’s lives, a brief, six-question Likert-scale survey for self-perceived Quality of Life (QOL) was also administered to study participants (Table 2). PUQE scores as well as QOL scores were collected on entry into the study and one-week after starting antiemetic therapy with promethazine, ondansetron, prochlorperazine, or metoclopramide. The patient PUQE and QOL scores were collected by telephone for patients unable to return for the one-week follow-up visit. A one-week follow-up was selected to try to capture the effects of the medications prescribed due to the fact that the condition of NVP is one that over time may resolve itself. If treatment with antiemetic medications had been started prior to enrollment, the subject was asked to provide a baseline PUQE score estimate retrospectively to the time treatment was initiated, then current PUQE score on day of enrollment and a follow-up PUQE score one week after enrollment. For these subjects, the baseline PUQE and QOL were used as the Initial PUQE and QOL scores. Final PUQE and QOL scores were taken at the one-week follow-up and a change in PUQE and QOL (Δ) were calculated by subtracting the initial score from the final score. For both the PUQE and QOL tools, higher scores indicate more negative symptoms and impact of NVP for the subject. The number of drugs prescribed for the women over the course of their treatment for NVP was also used as a surrogate for disease severity as most obstetric providers begin by prescribing one drug but prescribe more than one for recalcitrant or very severe cases.

**Table 1:** Motherisk Pregnancy Unique Quantification of Emesis (PUQE) scoring tool

**PUQE questions**	Score
1. In the last 24 hours, for how long have you felt nauseated or sick to your stomach?	
•Not at all (n = 1)	
•1 hour or less (n = 2)	
•2 to 3 hours (n = 3)	
•4 to 6 hours (n = 4)	
•More than 6 hours (n = 5)	
2. In the last 24 hours, how many times have you vomited or thrown up?	
•7 or more times (n = 5)	
•5 to 6 times (n = 4)	
•3 to 4 times (n = 3)	
•1 to 2 times (n = 2)	
•I did not throw up (n = 1)	
3. In the last 24 hours, how many times have you had retching or dry heaves without bringing anything up?	
•No times (n = 1)	
•1 to 2 times (n = 2)	
•3 to 4 times (n = 3)	
•5 to 6 times (n = 4) •7 or more times (n = 5)	
**Total Score**	

This survey scoring tool was administered at baseline and after a week of therapy.

**Table 2:** Impact of NVP on Normal Functioning Quality of Life (QOL) survey tool

	**Not at all**	**A little**	**Quite a bit**	**Very much**
1. My NVP has limited me in doing my work or other daily activities in the last week.	1	2	3	4
2. My NVP has limited me in pursuing my hobbies or other leisure time activities in the last week.	1	2	3	4
2. My NVP has prevented me from caring for my other children ________ I have no other children	1	2	3	4
4. My NVP has prevented me from eating in the last week.	1	2	3	4
5. My NVP has prevented me from taking prenatal vitamins in the last week.	1	2	3	4
6. My NVP has negatively affected my relationship with my partner in the last week.	1	2	3	4

Each of the ratings are totaled up for a total QOL score. NVP = Nausea and vomiting of pregnancy.

### Statistical analysis

All data were analyzed using SPSS-PASW Statistics v.18 (SPSS, Inc., Chicago, IL). Descriptive summary statistics were generated for maternal characteristics and the specific serotonin receptor genotype. In each case, the wild type alleles were compared to subjects having at least one variant allele. Linear regression modeling compared the correlation of genotype to the PUQE and QOL scores and number of drugs prescribed. Comparison of categorical variables was performed using chi-square testing. Continuous variables were compared using ANOVA testing or Mann–Whitney U or Kruskal Wallis tests for nonparametric data.

## Results

Twenty-nine women with NVP who had complete data and sampling and returned for their one week follow-up were included in this study. The mean age of subjects was 26.2 ± 5.3 years. The mean gestational age at presentation was 12.0 ± 1.4 weeks. The ethnic distribution of the subjects was 9 (32.1%) Caucasian, 17 (60.7%) African-American, and 2 (7.1%) Hispanic. Medications prescribed were promethazine, metoclopramide, prochlorperazine, and ondansetron. Some women 15/29 (51.7%) received more than one drug to control symptoms during the treatment period. No participants were using acupressure or were using non-prescription treatments such as ginger or vitamin B6 during the study time frame, although these were not specifically prohibited. Table 3 displays the NVP severity score summary for the study cohort. One woman’s DNA was unusable for genotyping, leaving 28 women for genetic analysis.

**Table 3:** Summary of NVP severity parameters for the study population

**Variable**	**Mean ± Standard deviation**	**Median**	**Range**
**Initial PUQE**	10.7 ± 3.4	12	3-15
**Final PUQE**	7.0 ± 3.5	6	2-15
**Change in PUQE**	4.7 ± 2.8	5	−2-9
**Initial QOL**	16.7 ± 4.2	17	8-23
**Final QOL**	11.9 ± 5.7	9	6-24
**Change in QOL**	5.3 ± 6.3	3	−4-16
**Number of drugs given**	1.6 ± 0.7	2	1-3

*PUQE* Pregnancy Unique Quantification of Emesis score. *QOL* Quality of Life score.

The serotonin receptor genotypes were obtained for all included subjects. For rs1176744, subjects were categorized as having the genotype AA (wild-type, 6, 21.4%), AC (heterozygous, n = 16, 57.1%), or CC (homozygous variant, n = 6, 21.4%). For rs3782025, subjects were homozygous wild type (AA, n = 3, 11.5%) or heterozygous (AG, n = 14, 23, 88.5%). For rs3831455, 22 subjects (84.6%) were homozygous wild type and 4 (15.4%) carried the deletion allele. For rs1062613 SNP, 10 (41.7%) were homozygous wild type (CC), 9 (37.5%) were heterozygous (CT), and 5 (20.8%) were homozygous variant alleles (TT).

**Figure 1:**
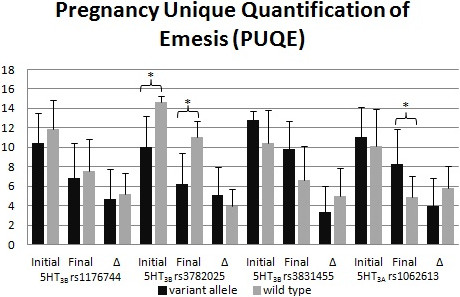
**Comparison of PUQE scores by serotonin receptor genotype.** Initial and final scores and the change (Δ) in the score are reported for 4 different single nucleotide polymorphisms. Given as Mean ± Standard Deviation, Significance; *, P ≤ 0.02.

**Figure 2:**
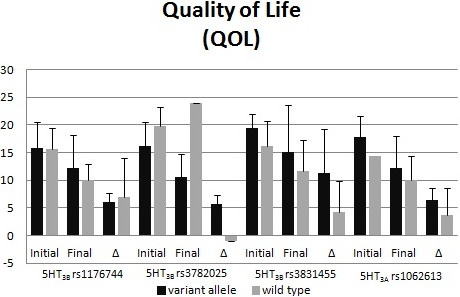
**Comparison of QOL scores by Serotonin receptor genotype.** Initial and final scores and the change (Δ) in the score are reported for 4 different single nucleotide polymorphisms. Given as Mean ± Standard Deviation.

Figures 1 and 2 display the polymorphisms’ impact on PUQE scores (Figure 1) and QOL scores (Figure 2). For the rs3782025 polymorphism, the initial and final PUQE scores were significantly better for carriers of a variant allele (p = 0.02 for both comparisons). The final PUQE score was significantly worse for carriers of the rs1062613 variant (p = 0.01). There were trends toward significant differences for rs3782025 final QOL scores (p = 0.08), rs3831455 final PUQE scores (p = 0.07), and rs1062613 initial QOL scores (p = 0.05).

Table 4 displays the difference in the mean number of drugs given to control NVP symptoms for the women with the different genotypes. Women with variant rs1176744 genotypes required treatment with a greater number of antiemetic drugs (p < 0.001). There were no significant differences in the percent of women who needed more than one drug to control symptom in any of the SNP comparison groups (data not shown).

**Table 4:** Comparison of number of drugs given to control NVP symptoms

**Serotonin receptor SNP RS ID#**	**Mean ± Std Dev wild type group**	**Mean ± Std Dev variant group**	**P value**
**rs1176744**	1.0 ± 0.0	1.7 ± 0.7	<0.001
(6 wild type, 22 variant)			
**rs3782025**	1.3 ± 0.6	1.6 ± 0.6	0.50
(3 wild type, 23 variant)			
**rs3831455**	1.6 ± 0.6	1.5 ± 1.0	0.73
(22 wild type, 4 variant)			
**rs1062613**	1.5 ± 0.5	1.6 ± 0.8	0.80
			(10 wild type, 14 variant)

## Discussion

Although the complete pathogenesis of NVP is unknown, serotonin is a key mediator in inducing nausea and vomiting through a mechanism in which 5-HT acts on its receptor in the gastrointestinal tract, 5-HT_3_. This receptor relays information to the central nervous system through depolarization of vagal afferent nerves and the vagus nerve. This signal is thought to initiate the vomiting reflex [19]. Excess serotonin is released by small intestinal enterochromaffin cells in response to drugs such as chemotherapeutic agents [20]. 5-HT_3_ antagonists are a class of medications that act at the 5-HT_3_ receptor, preventing serotonin from binding and thereby preventing vagal afferent stimulation and induction of the vomiting reflex. Many 5-HT_3_ antagonists are antiemetics and are used in the prevention and treatment of nausea and vomiting.

The ligand-gated ion channel serotonin receptor type 3 (5-HT_3_) is found in the autonomic, enteric, and sensory nervous systems [21]. Although five subunits of 5-HT_3_ have been identified in humans (5-HT_3A-3E_), 5-HT_3A_ is the only subunit that is capable of forming homo-oligomeric receptor complexes that are functional. All other subunits are able to form only heteropentameric receptor complexes with 5-HT_3A_[22]. Therefore, 5-HT_3A_ is a consistently important subunit and 5-HT_3B_ has previously been shown to be a major factor in HTR3 function [23]. Additionally, 5-HT_3A_ and 5-HT_3B_ are best characterized and both functional and clinically relevant genetic polymorphisms exist in the genes of these subunits so we focused on these relevant polymorphism. To our knowledge, only one other study has examined the relationship between NVP and 5-HT_3_ receptor genotype. This study focused largely on a different set of genetic polymorphisms and found a weak association between NVP and two *HTR3C* variants but did not find any association when examining two *HTR3A* variants, including one that was also tested in this study [16].

A variant form of the 5-HT_3B_ receptor (rs1176744 variants) has been shown to exhibit a substantially increased maximum response (7-fold increased mean open time) to serotonin compared to the wild type form [24]. While Tremblay et al. [14] found that this variant was not predictive of nausea and vomiting incidence in cancer patients, Sugai et al. [15] found this variant to be predictive of higher nausea incidence in a paroxetine-induced nausea setting. Our results indicate that this variant appears to contribute to decreased response to antiemetic therapy for NVP as homozygous variant subjects were prescribed more antiemetic medications to control symptoms than those with at least one wild type allele. These results suggest that women with this particular homozygous mutation in *HTR3B* may have more recalcitrant NVP. As such, homozygous variant patients may not be receiving high enough dosing of antiemetic therapy to inhibit serotonin binding at the 5-HT_3_ receptor target due to the increased affinity of the receptor for serotonin in these subjects.

The 5’-UTR *HTR3A* variant, rs1062613, has also been shown to have functional significance. This nonsense mutation occurs in the upstream open reading frame of *HTR3A.* Luciferase reporter constructs showed that presence of the variant allele results in significant increases in expression [25]. Despite this, Kaiser et al. [26], Sugai at al. [15], and Goeke et al. [16] found that *HTR3A* polymorphisms were not good predictors of antiemetic treatment in cancer patients, SSRI-induced nausea, or NVP, respectively. However, our data showed that despite statistically similar initial PUQE scores, carriers of a variant rs1062613 allele had significantly worse final PUQE scores. We also found that there is a trend toward significant differences in initial QOL scores with variant carriers having worse initial QOL compared to other patients. This discrepancy could be caused by medication and setting differences amongst studies. Our results suggest that patients who carry at least one variant allele may not be receiving a sufficient dose of antiemetics to antagonize the increased receptors that may be expressed.

Previous work has found that the 3 base pair insertion/deletion polymorphism, rs3831455, in the promoter region of the *HTR3B* gene was found to significantly associate with incidence of nausea and vomiting caused by chemotherapy [14]. This *HTR3B* promoter deletion, or variant, was also found to change the mRNA structure and was suggested to have an effect on translation efficiency [27] and nuclear protein binding [28]. Our results indicated a trend for this insertion/deletion polymorphism with worse final PUQE scores in patients carrying a variant allele. This could be due to increased translation and expression in those carrying the variant which could lead to insufficient antiemetic dosing to control symptoms.

Although the functional consequences of the *HTR3B* variant rs3782025 are unclear this polymorphism has been associated with vulnerability to alcohol use disorders (AUD), another condition that has been suggested to be affected by 5-HT dysfunction [29]. We found this polymorphism to be significantly associated with both initial and final PUQE scores, with variant carriers having better scores. Carriers of this variant also trended towards better final QOL scores. These data suggest that carrying a variant rs3782025 allele may be protective against NVP, as baseline severity and outcome are improved in these women.

## Conclusions

For patients with significant emesis this may be an important step in improving treatment strategies. If these findings are confirmed in larger studies, it is possible that women with certain serotonin receptor genotypes may be better suited for one antiemetic drug versus another or may require a higher starting dose of drug. Alternate therapy may be prescribed based on genotype or patients may start therapy on multiple drugs rather than monotherapy. This could provide significant time and money savings as well as provide much needed symptom relief and may improve safety for both the mother and the fetus.

Our study was limited by small sample size. This pilot study was not powered to find differences in secondary outcomes, however the trends seen here will inform samples size for follow-up studies. There was also a potential for recall bias, however validated measures were used, increasing the validity of the results. Another limitation is that the study did not mandate a specific NVP treatment algorithm and did not direct physician’s prescribing patterns in any way. As such, while the number of antiemetic medications given to each subject was used as a surrogate for severity of NVP, it is confounded by variability in provider practice. Because standard practice is to start with one drug and add more to obtain symptom control we believe that this is a reasonable surrogate of symptom severity. We also did not measure treatment adherence. These results need to be validated in a larger cohort. Additionally, ability to perform subgroup analyses of treatment groups by increasing sample size may improve the ability to build a pharmacogenetic treatment model, which could be used to guide clinical practice. A prospective treatment trial based on genotype and examining an expanded serotonin receptor pathway genetic analysis is currently being planned.

In conclusion, this pilot study of pharmacogenetic predictors of NVP therapy response demonstrates that genotype for serotonin receptor subunits 5-HT_3A_ and 5-HT_3B_ may play a role in disease severity and response. There is a need for more pharmacogenetic studies in pregnancy, particularly for conditions necessitating drug therapy such as NVP. As pharmacogenetic studies emerge in obstetric conditions such as NVP, the potential for individualized pharmacotherapy at the outset of treatment may be realized.

## Competing interests

Authors have no additional financial disclosures. The authors declare that they have no competing interests.

## Authors’ contributions

AL participated in genetic studies, analysis and manuscript drafts. JR participated in study design, analysis and manuscript drafts. CM participated in study design, implementation, and data acquisition. AT and CR participated in study design and analysis. DM conceived of the study and participated in the design and coordination of the study, analysis, and drafting of the manuscript. All authors have read and approved the final manuscript.

## Pre-publication history

The pre-publication history for this paper can be accessed here:

http://www.biomedcentral.com/1471-2393/13/132/prepub
